# Vertical sleeve gastrectomy-derived gut metabolite licoricidin activates beige fat thermogenesis to combat obesity

**DOI:** 10.7150/thno.81893

**Published:** 2023-05-21

**Authors:** Zhangliu Jin, Wen Meng, Ting Xiao, Jiangming Deng, Jing Wang, Jie Wen, Kai Chen, Liwen Wang, Juanhong Liu, Qingxin Li, Jieyu He, Zheng Wang, Wei Liu, Feng Liu

**Affiliations:** 1 Department of General Surgery, Division of Biliopancreatic and Metabolic Surgery, The Second Xiangya Hospital of Central South University, Changsha 410011, Hunan, China.; 2 National Clinical Research Center for Metabolic Diseases, Key Laboratory of Cardiometabolic Medicine of Hunan Province, and Metabolic Syndrome Research Center, The Second Xiangya Hospital of Central South University, Changsha 410011, Hunan, China.; 3 Department of Hepatology, Hunan Children's Hospital, Changsha 410000, Hunan, China.; 4 College of Bioscience & Biotechnology of Hunan Agricultural University, Changsha 410128, Hunan, China.

**Keywords:** Vertical sleeve gastrectomy, Gut metabolite, Licoricidin, Obesity, Thermogenesis

## Abstract

Obesity is a chronic metabolic disease, affecting individuals throughout the world. Bariatric surgery such as vertical sleeve gastrectomy (VSG) provides sustained weight loss and improves glucose homeostasis in obese mice and humans. However, the precise underlying mechanisms remain elusive. In this study, we investigated the potential roles and the mechanisms of action of gut metabolites in VSG-induced anti-obesity effect and metabolic improvement.

**Methods:** High-fat diet (HFD)-fed C57BL/6J mice were subjected to VSG. Energy dissipation in mice was monitored using metabolic cage experiments. The effects of VSG on gut microbiota and metabolites were determined by 16S rRNA sequencing and metabolomics, respectively. The metabolic beneficial effects of the identified gut metabolites were examined in mice by both oral administration and fat pad injection of the metabolites.

**Results:** VSG in mice greatly increased thermogenic gene expression in beige fat, which was correlated with increased energy expenditure. VSG reshaped gut microbiota composition, resulting in elevated levels of gut metabolites including licoricidin. Licoricidin treatment promoted thermogenic gene expression in beige fat by activating the Adrb3-cAMP-PKA signaling pathway, leading to reduced body weight gain in HFD-fed mice.

**Conclusions:** We identify licoricidin, which mediates the crosstalk between gut and adipose tissue in mice, as a VSG-provoked anti-obesity metabolite. Identification of anti-obesity small molecules should provide new insights into treatment options for obesity and its associated metabolic diseases.

## Introduction

Obesity, which is related with various metabolic and cardiovascular diseases as well as certain types of cancer, has become one of the most serious public health crises worldwide 1,2. Obesity is caused by imbalanced nutrient input and energy expenditure, which leads to excessive ectopic fat deposition in metabolically important organs such as adipose tissue and liver, resulting in various medical disorders such as coronary heart disease and type 2 diabetes 3,4. Current treatments for obesity include calorie control, physical activity, weight-loss pharmacological medications, and bariatric surgery 5,6. Bariatric surgery appears to be the safest and the most effective clinical intervention for the treatment of obesity to date 7. In addition to weight loss, bariatric surgery produces remission of type 2 diabetes and hypertension, as well as decreased risk of developing certain types of cancer 8,9.

Vertical sleeve gastrectomy (VSG), which improves obesity and glucose homeostasis in both rodent and human studies 10,11, is the most commonly performed bariatric procedure in the clinic in the United States 12. Some earlier studies suggest that VSG improves obesity by restricting food intake and interfering with digestion (restrictive/malabsorptive) 13. However, more recent studies demonstrate that many of the beneficial effects of bariatric surgery are mediated by other mechanisms such as changes in bile acid metabolism, central regulation of metabolism, and alteration in gut microbiota 14,15. Consistent with this view, fecal microbiota transplantation using feces from VSG mice in germ-free mice improves glucose metabolism 16. Antibiotic-induced disruption of intestinal microbiota abrogates the metabolic benefits of VSG 17. Moreover, clinical investigations showed that fecal transplantation from patients after bariatric surgery confers a beneficial effect on patients with obesity 18. However, the precise mechanisms underlying the metabolic benefits of gut microflora in VSG remain unclear.

Brown adipose tissue (BAT) and beige fat have emerged as major regulators of energy homeostasis owing to their ability to burn glucose and fat to produce heat 19-21. Numerous studies have demonstrated that enhanced non-shivering thermogenesis in BAT and beige fat increases energy expenditure and subsequently counteracts obesity 22,23. Interestingly, data from preclinical and clinical studies reveal that VSG could enhance beige fat thermogenesis 24, suggesting a mechanism by which VSG improves obesity and its associated metabolic dysfunction. However, how VSG promotes beige fat thermogenesis remains obscure.

In this study, we show that VSG promotes beige fat thermogenesis and inhibits high-fat diet (HFD)-induced body weight gain in mice. The effect of VSG is independent of the activation of the sympathetic nervous system (SNS), but depends on the gut metabolite licoricidin, which is upregulated by VSG in the intestine and serum. Treating mice with licoricidin promotes beige fat thermogenesis and protects mice against HFD-induced obesity. Mechanistically, licoricidin stimulates thermogenic gene expression in adipocytes by activating the Adrb3-cAMP-PKA signaling pathway. Our study uncovers a new mechanism by which VSG suppresses obesity in mice and identifies licoricidin as a potential target for developing an effective anti-obesity therapeutic treatment.

## Materials and Methods

### Experimental animals

Male C57BL/6J wild-type mice were purchased from Slac Laboratory Animal Inc. (Shanghai, China) at seven-week-old. All animals have received water and a normal chow ad libitum after birth. After a week of acclimatization, mice were randomly allocated to two cohorts: VSG and sham operation. Thereafter, an HFD (Research Diets Inc., NJ. USA) and water were available to induce obesity in mice for 12 weeks and subsequently subjected to VSG. All animals were housed under 12/12-h light-dark cycle at room temperature (21±1 °C). This investigation was approved by the Institutional Animal Care and Use Committee of Central South University (Permission number: CSU2020406) and all applicable institutional guidelines for animal care and use were executed.

### Mouse body weight, food intake, body composition, and fecal energy density

Mouse body weight was monitored weekly at 10:00-12:00 h. Mouse food intake was determined on a weekly basis. Total daily food intake was assessed by dividing the amount of food left by the number of mice in each cage. Body composition was measured using Bruker's minispec LF50 BCA-Analyzer (Karlsruhe, Germany). Mouse feces were collected, dried, and ground into a powder in preparation for detection. Fecal energy density was determined using an oxygen bomb calorimeter (IKA, Germany) according to a previously described method 25.

### Glucose and insulin tolerance test

All animals were fasted for 16 h before the glucose tolerance test (GTT) and then intraperitoneally injected with D-glucose (1.5 g/kg body weight). All animals were fasted for 4 h before insulin tolerance test (ITT) and then intraperitoneally injected with human insulin (0.75 U/kg body weight). Blood glucose levels were monitored in the tail blood at 0, 15, 30, 60, 90, and 120 min after glucose or insulin administration using an Accu-Chek Performa glucometer (Roche, Switzerland).

### Hematoxylin-eosin (H&E), Oil-Red O staining, and histological analysis

For H&E staining, after tissues harvested, adipose tissues, liver, and the intestine were fixed using 4% paraformaldehyde for 24 h and subsequently embedded in paraffin. Tissues were sectioned into 5 μm-thick sections, followed by H&E staining. For Oil-Red O staining, the fixed tissues were embedded in OCT and promptly frozen. Tissue sections (10 μm thick) were warmed up and incubated using 0.3% Oil-Red O staining solution, followed by washing several times with water. For fat cell quantification, the average diameters of the adipocytes were measured using the Image J software.

### Surgery

All animal procedures were performed under aseptic conditions and anesthetized with isoflurane. A 1.0-cm laparotomy incision was made and then the stomach was slightly hauled back to adequately expose the whole stomach and distal esophagus, followed by the ligation and cutting of the gastrosplenic ligate and vessels. Two clips were placed on the stomach approaching the lesser curvature, removing approximately 80% of the stomach, and leaving a tubular remainder. The surplus stomach was uninterruptedly sutured with 7-0 nylon monofilament (Ethicon, USA) and then the abdominal incision was closed using a 3-0 silk braided non-absorbable suture (Ethicon, USA). In the sham group, mice underwent gastric cut and re-anastomosis in situ, and the operating time was prolonged to the same time as the VSG mice. After surgery, all mice were injected with 1.0 mL 0.9% saline and 1.0 mg/kg buprenorphine and subsequently set on a heating pad to expedite revival. Mice received a liquid diet (ENSURE, Zwolle, Netherlands) for 48 h after surgery and were then switched to an HFD.

### Serum licoricidin assay

Blood samples were collected and centrifuged in preparation for analysis. The concentration of licoricidin in mouse serum was measured by high-performance liquid chromatography (HPLC) (Agilent Technologies, USA) as previously described 26.

### Antibiotics treatment

The wide-spectrum antibiotics cocktail (ABX, neomycin 1 g/L, metronidazole 1g/L, vancomycin 0.5 g/L, ampicillin 1g/L) was administered by drinking water for 2 weeks and subsequently prepared for further surgery, as previously described 27. All antibiotics were purchased from Sigma-Aldrich.

### Bilateral denervation of subcutaneous fat pad

Diet-induced obese (DIO) mice were anesthetized with isoflurane and then the bilateral incisions were made approximately 0.5 cm. 6-hydroxydopamine (6-OHDA, Sigma-Aldrich, USA) was dissolved in PBS containing 1% ascorbic acid and subsequently injected into the bilateral subcutaneous fat pads (2 μL for per injection, 24 μL per pad), according to previously described 28. The same volume of vehicles was injected into control mice. The bilateral incisions were closed as described above. The VSG and Sham mice were immediately prepared for surgery.

### Quantitative real-time PCR

All RNAs of samples were extracted using TRIzol Reagent (Invitrogen, USA). RNA (1 μg) was converted to cDNA synthesis (Thermo Fisher Scientific, USA). Quantitative real-time PCR was carried out with the SYBR green mix (Roche, Switzerland) and quantitated using the ViiA7 System (Life Technology, USA). The primer sequences for these genes are displayed in Table S1.

### Western blot

For western blot analysis, antibodies against UCP1, PGC1α, Occludin, P-PKA-substrate, CREB, P-CREB, tyrosine hydroxylase (TH), and PDE4D were purchased from Cell Signaling Technologies (CST, USA). Antibodies against β3-adrenergic receptor (β3-AR), PDE3B, and PDE4B were purchased from Abcam (Cambridge, UK). All protein levels were normalized to β-actin levels (Sigma-Aldrich, USA).

### Indirect calorimetry and intestinal permeability assay

Each mouse was placed in the Comprehensive Lab Animal Monitoring System (CLAMS, USA) for 24 h to accommodate the new surroundings. Oxygen consumption and energy expenditure of each mouse in live-in cages were monitored for 48 h. Physical activity monitoring was carried out with metabolic parameters using the CLAMS. Intestinal permeability was measured by a fluorescence spectrophotometer as described previously 29.

### Oral administration of licoricidin

Each group of C57BL/6J mice was fed an HFD for 12 weeks. Licoricidin was dissolved in 0.9% saline containing 0.5% carboxymethylcellulose sodium (CMC, Sigma-Aldrich, USA). Licoricidin (10 mg/kg, Chemfaces, Wuhan, China) was administered daily by oral gavage during the indicated time. Vehicle mice in the control group were daily given the same amount of 0.9% saline containing 0.5% CMC by oral gavage without licoricidin. The weight of each mouse was monitored daily. After 4 weeks, the mice were euthanized and then tissues were harvested.

### Fat pad injection

Licoricidin was dissolved in PBS containing 2-hydroxypropyl β-cyclodextrin (2-Hp-β-CD, Sigma-Aldrich, USA). The DIO mice were anesthetized with isoflurane and then licoricidin (10 μg/kg) was injected daily into the bilateral inguinal fat pads using a microsyringe (Smiths Medical ASD, Inc., Keene, NH, USA), according to previously described 30. The same volume of PBS containing 2-Hp-β-CD was injected daily into vehicle mice in the control group without licoricidin. The weight of each mouse was monitored daily.

### Primary cell culture

Subcutaneous adipose tissues were collected and cultured following the protocol as described 31. In brief, subcutaneous fat tissues isolated from 2-week-old male C57BL/6J mice were promptly minced and then digested with type II collagenase (Sigma-Aldrich, USA). The digested fat tissue was filtered using a nylon screen (100 μm) and then centrifuged at 2500 rpm for 5 min at 4 °C. Cells were seeded in a 10-cm dish. The differentiation of white adipocytes was carried out according to previously described methods 32.

### *In vitro* licoricidin treatment

To explore the impact of licoricidin on thermogenesis, differentiated mature mouse primary subcutaneous adipocytes were treated with vehicle and different doses of licoricidin for 24 h. For siRNA transfection, siRNA (250 nmol/L) was transfected into differentiated primary subcutaneous adipocytes using the Lipofectamine transfection reagent (Thermo Fisher Scientific, USA). 8 h later, the cells were then treated with vehicle or licoricidin (0.1 μM) for 24 h.

### ELISA

cAMP levels in mouse primary subcutaneous adipocytes were measured according to the manufacturer's protocol of a cAMP ELISA kit (Cell Biolabs, USA), as previously described 33.

### Microscale thermophoresis (MST) assay

The biophysical parameters of interaction between licoricidin and β3-AR were measured by the MST assay, as previously described 34.

### Confocal microscopy

To determine the cellular localization of licoricidin, biotin was used to label licoricidin. Differentiated adipocytes were treated with biotin-labeled licoricidin (1 μM) for 2 h and then fixed for 30 min. Cells were rinsed twice with PBS and subsequently incubated with Streptavidin (BioLegend, San Diego, CA, USA) for 30 min. Cells were rinsed twice with PBS and then incubated with phalloidin (BioLegend, San Diego, CA, USA) and DAPI (BioLegend, San Diego, CA, USA) for 10 min, respectively. Confocal microscopy images were taken on a laser scanning microscope (Zeiss, Germany).

### Immunoblotting

HEK293T cells were cultured in DMEM supplemented with 10% fetal bovine serum (FBS). HEK293T cells transfected with FLAG-tagged β3-AR were lysed at 24 h after transfection. Biotin-labeled licoricidin was gently rotated with and NeutrAvidin beads (Thermo Fisher Scientific, USA) for 7 h and subsequently incubated with transfected cell lysates overnight. The prepared samples were subjected to immunoblotting with an antibody against the FLAG tag.

### Statistical analysis

Statistical analysis of the data was carried out using Student's t-test, ANCOVA or ANOVA. The P ≤ 0.05 level was considered to be statistically significant. GraphPad Prism 8.4.2 (GraphPad, San Diego, CA, USA) and SPSS were used to analyze all results. All results are displayed as mean ± SEM and are representative of at least three independent experiments.

## Results

### VSG promotes beige fat thermogenesis and energy expenditure by a sympathetic nerve system-independent mechanism in HFD-induced obese mice

**Figure 1:**
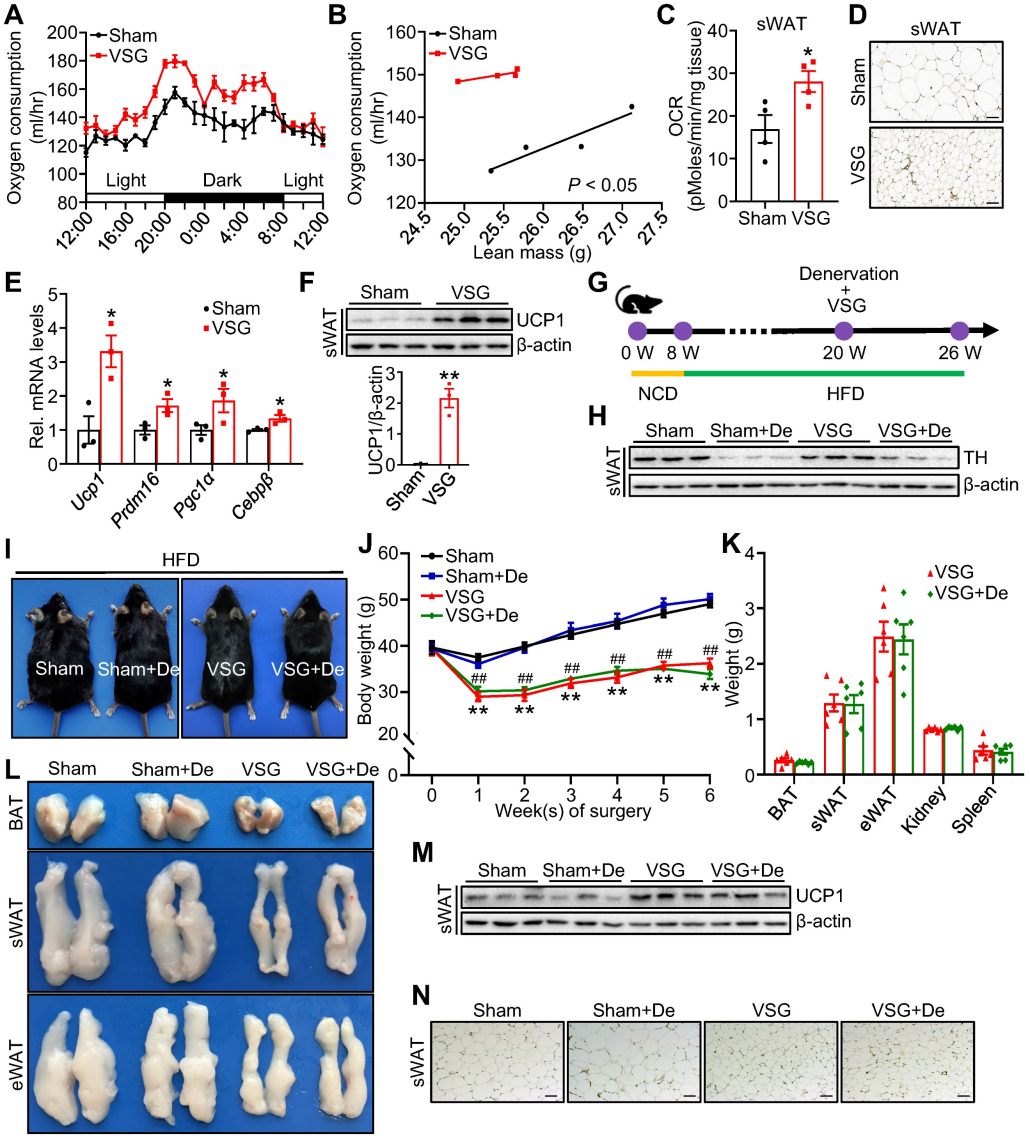
**VSG promotes beige fat thermogenesis and energy expenditure by a sympathetic nerve system-independent mechanism in HFD-induced obese mice.** C57BL/6J mice fed high-fat diet (HFD) induced obesity for 12 weeks and subsequently were subjected to vertical sleeve gastrectomy (VSG). (A) The oxygen consumption of VSG and Sham mice was examined by indirect calorimetry using the CLAMS (*n* = 4 mice per group). CLAMS, comprehensive lab animal monitoring system. (B) The regression of oxygen consumption with lean mass in VSG and Sham mice (*n* = 4 mice per group). (C) Basal oxygen consumption rate (OCR) of isolated sWAT of VSG and Sham mice was measured by Seahorse (*n* = 4 mice per group). sWAT, subcutaneous adipose tissue. (D) Immunohistochemical staining of uncoupling protein 1 (UCP1) in sWAT-derived from VSG and Sham mice 8 weeks post-surgery. Scale bar, 100 µm. (E) mRNA expression of thermogenic genes in sWAT of VSG and Sham mice 8 weeks post-surgery. Prdm16, PR domain-containing 16. Pgc1α, peroxisome proliferator-activated receptor gamma coactivator 1-alpha. Cebpβ, CCAAT enhancer binding protein beta. (F) UCP1 protein expression in sWAT of VSG and Sham mice 8 weeks post-surgery. (G) Experimental timeline. NCD, normal chow diet. (H) Tyrosine hydroxylase (TH) protein expression in sWAT of Sham, Sham + De (denervation), VSG, and VSG + De mice 6 weeks post-surgery. (I) Representative images of Sham, Sham + De, VSG, and VSG + De mice 6 weeks post-surgery. (J) Body weight of Sham (*n* = 5), Sham + De (*n* = 5), VSG (*n* = 6), and VSG + De (*n* = 6) mice 6 weeks after surgery. * Sham vs. VSG; # Sham + De vs. VSG + De. (K) Weights of sWAT, eWAT, BAT, and other tissues of VSG and VSG + De mice (*n* = 6 mice per group). eWAT, epididymal adipose tissue. BAT, brown adipose tissue. (L) Representative images of BAT, sWAT, and eWAT derived from Sham, Sham + De, VSG, and VSG + De mice. (M) UCP1 protein expression in sWAT of Sham, Sham + De, VSG, and VSG + De mice 6 weeks post-surgery. (N) Immunohistochemical staining of UCP1 in sWAT-derived from Sham, Sham + De, VSG, and VSG + De mice. Scale bar, 100 µm. All data are means ± SEM. Statistical values *p < 0.05, **p < 0.01, ##*p* < 0.01 are analyzed by two-way ANOVA, ANCOVA (B), or Student's *t*-test.

To determine the potential mechanism by which VSG improves metabolism, we performed VSG on DIO mice (Figure S1A-D). Compared to the Sham control mice, the VSG mice displayed a great reduction in fat mass (Figure S1E). VSG also markedly alleviated diet-induced hepatosteatosis (Figure S1F) and significantly improved glucose tolerance (Figure S1G) as well as insulin sensitivity (Figure S1H) in mice. The VSG mice showed similar daily food intake (Figure S1I), fecal energy density (Figure S1J), and physical activity (Figure S1K) after a 3-week recovery period compared to the Sham mice. The decreased food intake in the first 2 weeks did not contribute to VSG-induced long-term weight loss (Figure S1L). However, the VSG mice displayed a significantly increased energy and oxygen expenditure during the total 24-hour period (Figure 1A-B and Figure S1M-N) and basal oxygen consumption rate (OCR) in subcutaneous adipose tissue (sWAT) (Figure 1C), suggesting that VSG may reduce body weight gain by increasing energy expenditure. In line with this view, VSG markedly induced beige fat thermogenesis as demonstrated by increased multilocular adipocytes (Figure 1D), elevated expression of thermogenic genes such as *Ucp1*, *Prdm16*, *Pgc1α,* and *Cebpβ* (Figure 1E), and increased UCP1 protein levels (Figure 1F) in mouse sWAT. However, no significant difference in UCP1 protein levels were observed in BAT and epididymal adipose tissue (eWAT) of the VSG mice compared to the Sham control mice (Figure S1O). Altogether, these findings demonstrate that VSG may reduce obesity by increasing beige fat thermogenesis and energy expenditure.

Activation of the SNS plays a crucial role in regulating thermogenic activity in adipose tissue 35. To examine whether the promoting effect of VSG on beige fat thermogenesis depends on SNS activation, we performed denervation experiments on VSG mice (Figure 1G-H). No significant differences in mice body size, body weight gain, weight of fat pad (Figure 1I-K), fat and liver lipids were observed between the denervated VSG mice and control VSG mice (Figure S1P-Q). Additionally, denervation had no significant effect on UCP1 protein expression in sWAT of VSG mice (Figure 1M-N). These findings suggest that VSG promotes beige fat thermogenesis through an SNS-independent mechanism.

### Licoricidin in gut and serum is increased following VSG

Compelling evidence suggests a link between the anti-obesity effect of VSG and changes in gut microbial communities 36. To characterize the underlying mechanism, we amplified 16S rRNA from the microbiome isolated from the intestine of Sham and VSG mice. The β diversity analysis showed that the ingredients and relative abundance of the microflora were notably different between the VSG and Sham groups (Figure S2A). Operational taxonomic units (OTUs) analysis also revealed a difference in sample diversity between the VSG and Sham mice (Figure S2B). Hierarchical clustering of individual genus confirmed that VSG altered intestinal microflora in mice (Figure S2C). By relative abundance analysis, we found that VSG significantly increased the abundance of the phylum *Firmicutes* (Figure S2D), but significantly reduced the abundance of *Bacteroidetes* (Figure S2E) in mice. Consistent with these results, the linear discriminant analysis (LDA) effect size (LEfSe) study revealed that the phylum *Firmicutes* was the prominent composition of intestinal microbiota in the VSG mice (Figure S2F). Kyoto encyclopedia of genes and genomes (KEGG) function analysis revealed the upregulation of several pathways such as those related to xenobiotics biodegradation and metabolism, lipid metabolism, infectious diseases, and other amino acid metabolism in the VSG mice compared to the Sham control mice (Figure S2G), suggesting that alterations in these pathway-related functions may contribute to the improved metabolic effects. To test whether altered microbiota has any effect on the status of the gut, we examined the mRNA expression of inflammatory cytokines in the small intestine and colon of VSG mice and Sham control mice. While VSG had no significant effect on the expression of *Il-6* and *Tnfα* in the mouse small intestine and colon, it significantly reduced the mRNA expression of *Il-1β* (Figure S3A-B), suggesting that VSG reduced intestinal inflammation in mice. VSG also greatly increased the protein levels of Occludin, a tight junction protein, in the mouse small intestine and colon (Figure S3C). Consistent with this result, the VSG mice showed improved intestinal integrity (Figure S3D) and permeability (Figure S3E) compared to the Sham mice.

**Figure 2:**
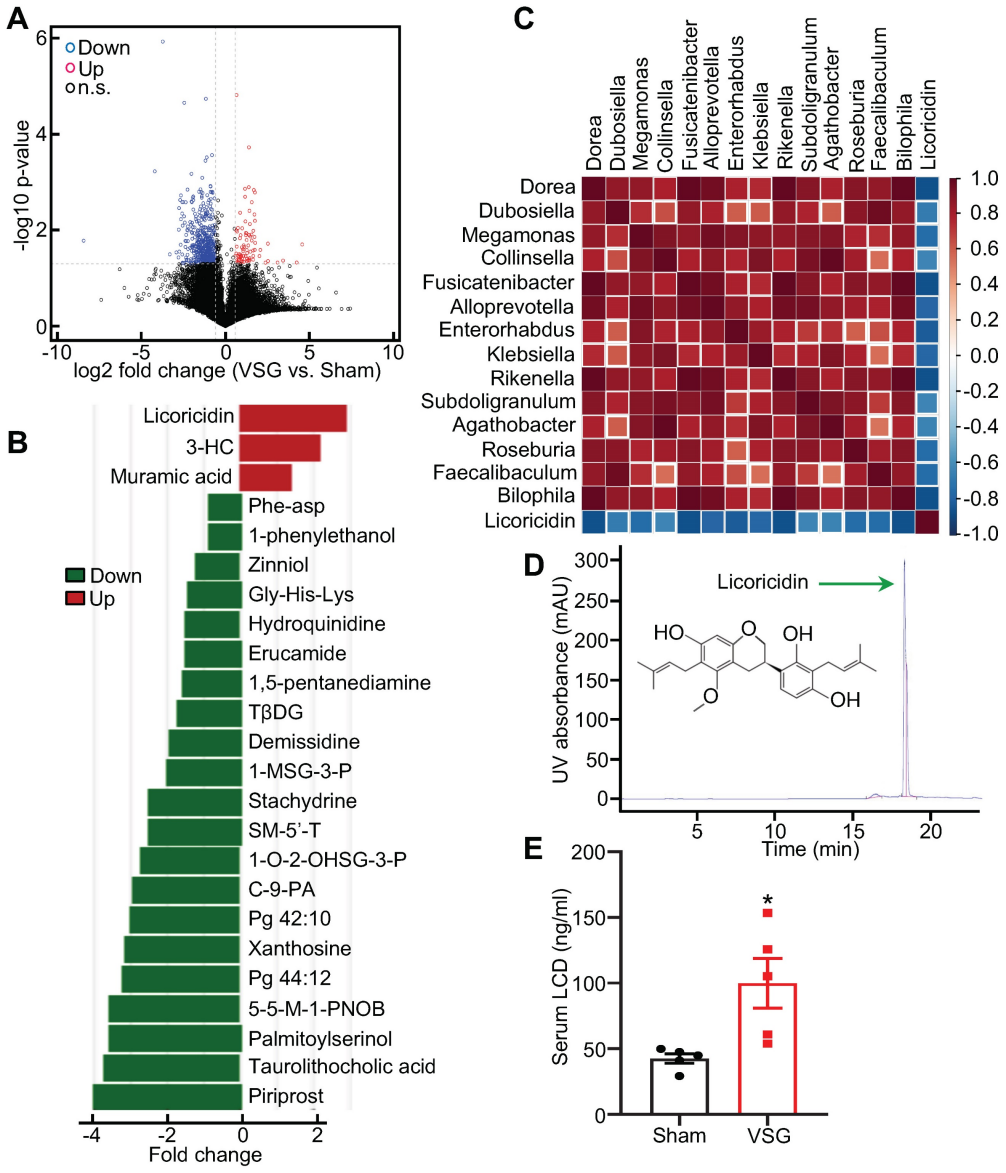
** Licoricidin in gut and serum is increased following VSG.** (A) Volcano plot of metabolomics in the Sham (*n* = 3) and VSG (*n* = 4) groups. The graph shows the average log2 ratios of abundances between Sham and VSG groups for each individual species and the corresponding p values. Red circle represents up-regulated metabolites (FC > 1.5 and *p* value < 0.05). Blue circle represents down-regulated metabolites (FC < 0.67 and *p* value < 0.05). Black circle represents not differentially expressed metabolites. Up, up-regulate; Down, down-regulate; n.s., not significant. (B) Histogram of the differentially expressed metabolites in Sham (*n* = 3) and VSG (*n* = 4) groups. Differentially expressed metabolites were found to be significant with predictive variable important in the projection (VIP) > 1 and *p* < 0.05 by analysis of variance and orthogonal projection to latent structure discriminant analysis (OPLS-DA). 3-HC, 3-hydroxybutyryl carnitine. TβDG, Thymol-beta-d-glucoside. 1-MSG-3-P, 1-myristoyl-sn-glycero-3-phosphocholine. SM-5'-T, S-methyl-5'-thioadenosine. 1-O-2-OHSG-3-P, 1-o-hexadecyl-2-o-(5z,8z,11z,14z,17z-eicosapentaenoyl)-sn-glyceryl-3-phosphorylcholine. C-9-PA, Cis-9-palmitoleic acid. 5-5-M-1-PNOB, 5-(diethoxyphosphoryl)-5-methyl-1-pyrroline-n-oxide-biotin. (C) Matrix graph of differentially expressed microflora and licoricidin in Sham (*n* = 3) and VSG (*n* = 4) groups by spearman analysis. Top, microflora. (D) Licoricidin (LCD) was measured by high-performance liquid chromatography (HPLC). (E) The LCD concentration in mouse serum following surgery was assessed (*n* = 5 mice per group). All data are means ± SEM. Statistical values *p < 0.05, **p < 0.01 are analyzed by Student's *t*-test between Sham and VSG groups.

To determine the potential mechanism by which altered intestinal microenvironment mediates VSG-induced beige fat thermogenesis, we performed metabolomics analysis of feces derived from Sham and VSG mice. The volcano plot of the mass spectrum data showed that a wide range of gut metabolites was altered in the VSG mice compared to the Sham control mice (Figure 2A). Among these differentially expressed molecules, licoricidin, muramic acid, and 3- hydroxybutyryl carnitine (3-HC), were markedly upregulated in the gut of the VSG mice compared to the Sham control mice (Figure 2B). Spearman correlation and matrix graph analysis revealed that licoricidin was negatively correlated with *Dorea, Fusicatenibacter, Subdoligranulum,* and* Bilophila* (Figure 2C), a group of harmful microbes involved in metabolic disease 37. Notably, serum licoricidin levels measured by HPLC were greatly increased in the VSG mice compared to the Sham control mice (Figure 2D-E and Figure S2H).

### Antibiotics treatment abrogates metabolic improvement after VSG

**Figure 3:**
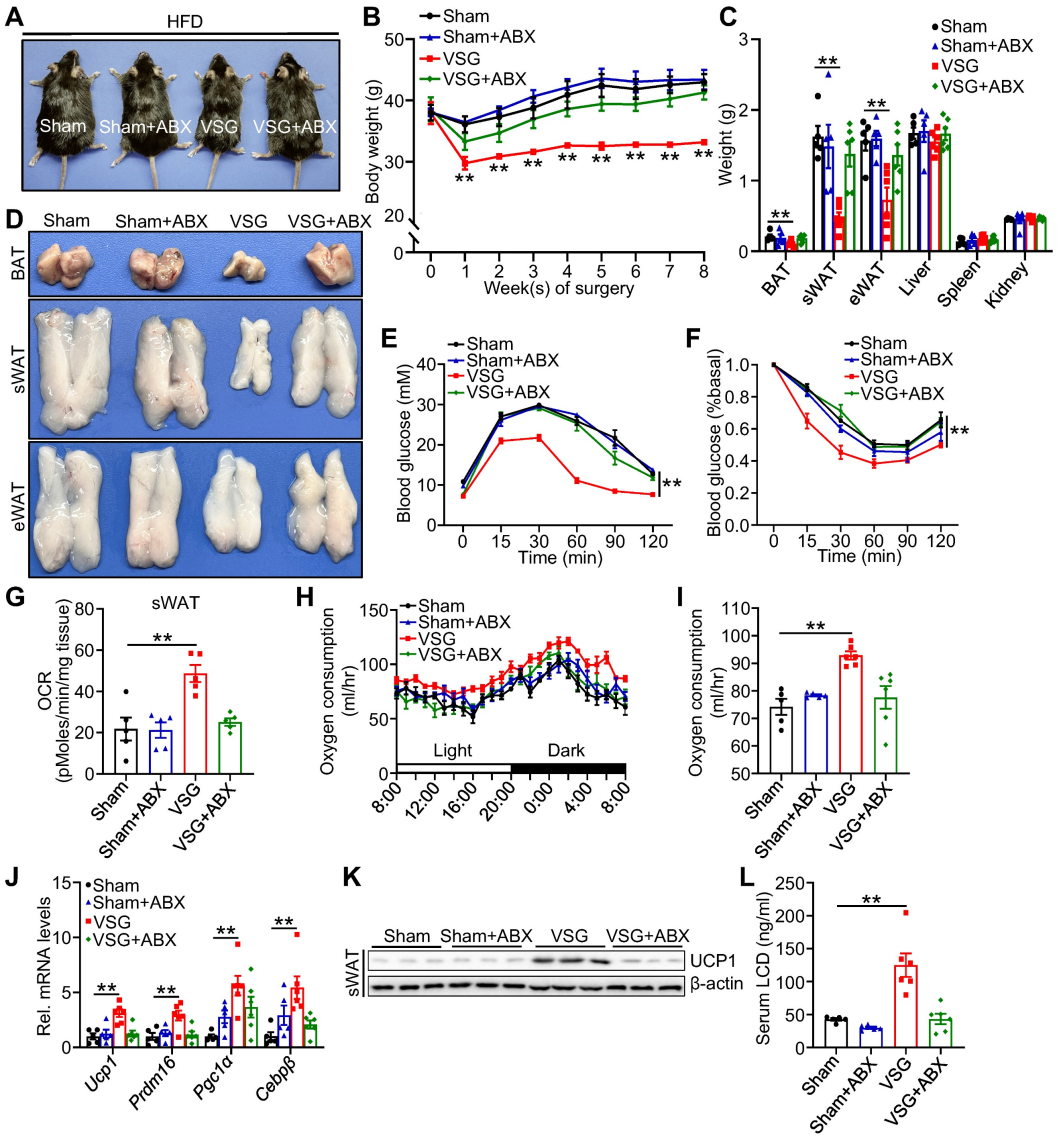
** Antibiotic exposure abrogates weight loss and beige fat thermogenesis following VSG.** (A) A representative image of Sham, Sham + ABX, VSG, and VSG + ABX mice 8 weeks post-surgery. (B) Body weight of Sham (*n* = 5), Sham + ABX (*n* = 5), VSG (*n* = 6), and VSG + ABX (*n* = 6) mice 8 weeks after surgery. * Sham vs. VSG. (C) Weights of sWAT, eWAT, BAT and other tissues of Sham (*n* = 5), Sham + ABX (*n* = 5), VSG (*n* = 6), and VSG + ABX (*n* = 6) mice. * Sham vs. VSG. (D) Representative images of BAT, sWAT, and eWAT derived from Sham, Sham + ABX, VSG, and VSG + ABX mice. (E) Glucose tolerance test was executed on Sham (*n* = 5), Sham + ABX (*n* = 5), VSG (*n* = 6), and VSG + ABX (*n* = 6) mice. * Sham vs. VSG. (F) Insulin tolerance test was executed on Sham (*n* = 5), Sham + ABX (*n* = 5), VSG (*n* = 6), and VSG + ABX (*n* = 6) mice. * Sham vs. VSG. (G) Basal OCR of isolated sWAT of Sham, Sham + ABX, VSG, and VSG+ ABX mice was assayed by Seahorse. (H) The oxygen consumption of Sham (*n* = 5), Sham + ABX (*n* = 5), VSG (*n* = 6), and VSG + ABX (*n* = 6) mice was examined. (I) The average of oxygen consumption of Sham (*n* = 5), Sham + ABX (*n* = 5), VSG (*n* = 6), and VSG + ABX (*n* = 6) mice. (J) The expression of thermogenic genes was measured by quantitative real-time PCR (qPCR). (K) The protein level of UCP1 was detected by western blot. (L) The LCD concentration in mouse serum following surgery was assessed (*n* = 5-6 mice per group). All data are means ± SEM. Statistical values *p < 0.05, **p < 0.01 are analyzed by two-way ANOVA, ANCOVA (I), or Student's *t*-test.

To further validate whether the shifts of gut microenvironment contribute to metabolic benefits imparted by VSG, ABX administration eliminated gut microbiota and then prepared for VSG. Relative to Sham+ABX mice, ABX administration markedly blunted body weight loss, weight reduction of fat pad, glucose tolerance, and insulin sensitivity (Figure 3A-F), and beige fat thermogenesis (Figure 3G-K) in VSG+ABX mice. Moreover, ABX administration greatly decreased serum licoricidin levels after VSG (Figure 3L). Overall, these findings suggest that licoricidin may contribute to VSG-induced weight loss in obese mice.

### Licoricidin ameliorates obesity and insulin resistance by promoting beiging fat thermogenesis

**Figure 4:**
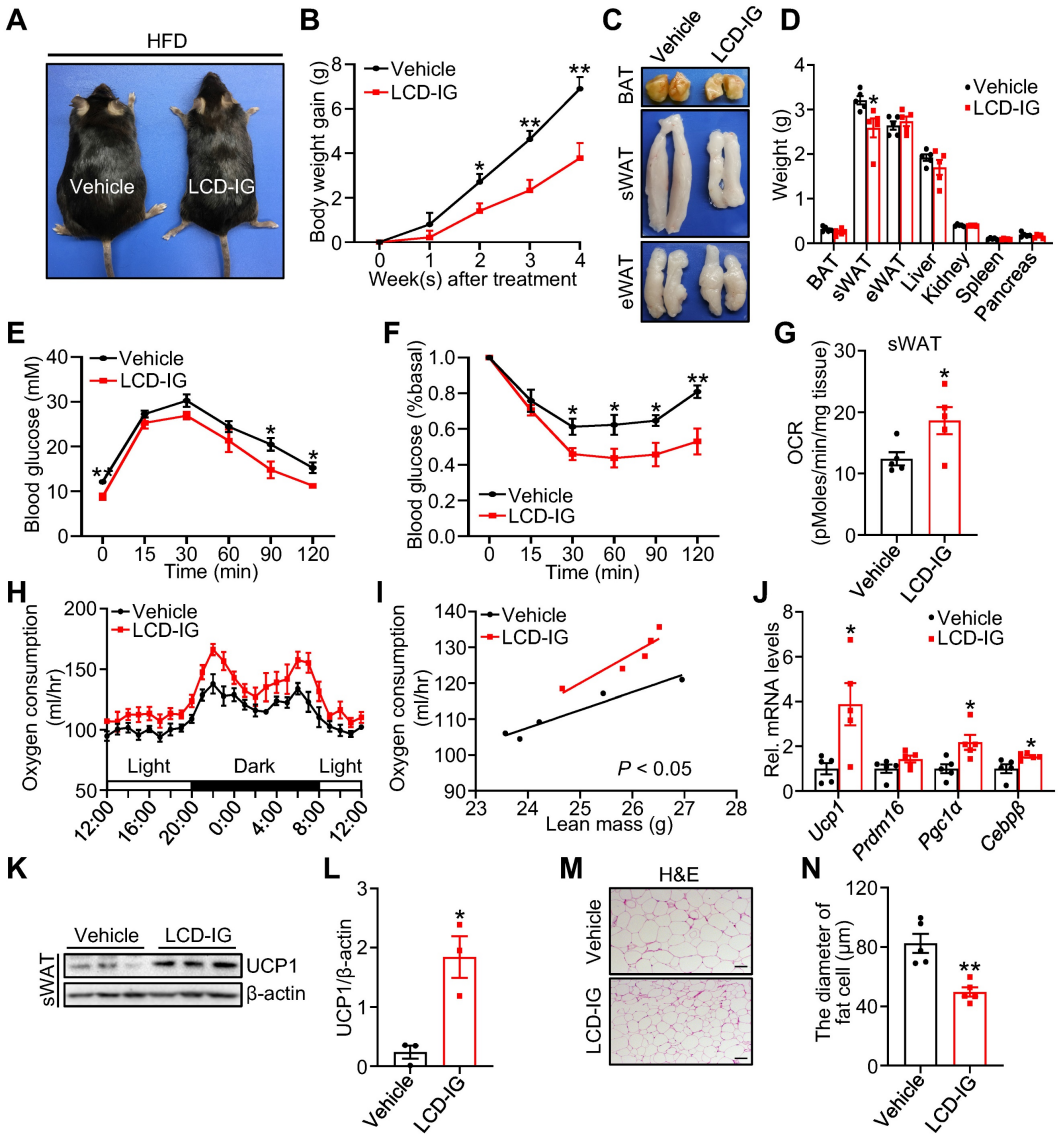
** Licoricidin ameliorates obesity and insulin resistance by promoting beiging fat thermogenesis.** (A) A representative image of HFD mice after LCD treatment. HFD mice were treated daily with LCD (10 mg/kg) by intragastric administration (LCD-IG) for 4 weeks (*n* = 5 mice per group). (B) Body weight gain of HFD mice during LCD treatment. (C) Representative photos of BAT, sWAT, and eWAT derived from vehicle and LCD-IG mice. (D) Weights of sWAT, eWAT, BAT, and other tissues of vehicle and LCD-IG mice (*n* = 5 mice per group). (E) Glucose tolerance test was executed on vehicle and LCD-IG mice (*n* = 5 mice per group). (F) Insulin tolerance test was executed on vehicle and LCD-IG mice (*n* = 5 mice per group). (G) Basal OCR of isolated sWAT of vehicle and LCD-IG mice was assayed by Seahorse (*n* = 5 mice per group). (H) The oxygen consumption of vehicle and LCD-IG mice (*n* = 5 mice per group) was examined. (I) The regression of oxygen consumption with lean mass in vehicle and LCD-IG mice (*n* = 5 mice per group). (J) The expression of thermogenic genes was measured by qPCR (*n* = 5 mice per group). (K) The protein level of UCP1 was detected by western blot. (L) Analysis of the gray image of UCP1 protein in sWAT between vehicle and LCD-IG groups. (M) H&E of sWAT-derived from vehicle and LCD-IG mice. Scale bar, 100 µm. (N) sWAT adipocytes diameters in vehicle and LCD-IG mice were quantified (*n* = 5 mice per group). All data are means ± SEM. Statistical values *p < 0.05, **p < 0.01 are analyzed by Student's *t*-test, two-way ANOVA, or ANCOVA (I) between vehicle and LCD-IG groups.

To directly test whether licoricidin has an anti-obesity effect, we administered DIO mice with licoricidin (10 mg/kg) by daily gavage for 4 weeks. Licoricidin treatment markedly decreased mouse body size (Figure 4A), body weight gain (Figure 4B), subcutaneous fat pad size (Figure 4C), and sWAT weight (Figure 4D), but had little effect on the weight of other tissues such as BAT, eWAT, liver, kidney, spleen, and pancreas (Figure 4D). Compared to control mice, the licoricidin-treated mice displayed a great improvement in both glucose tolerance (Figure 4E) and insulin sensitivity (Figure 4F). Together, these results indicate that licoricidin plays a beneficial role in alleviating HFD-induced adiposity and metabolic disorders.

To elucidate the mechanism by which licoricidin treatment suppressed mouse body weight gain, we examined the energy expenditure of the subcutaneous fat pad by using an XF-24 analyzer. We found that licoricidin-treated mice had significantly higher OCR and oxygen consumption compared with the vehicle-treated mice (Figure 4G-I). In line with the increased oxygen consumption, the mRNA (Figure 4J) and protein (Figure 4K-L) levels of several thermogenic genes were greatly induced in sWAT of the licoricidin-treated mice compared to vehicle-treated mice, concurrently with reduced adipocyte cell size (Figure 4M-N). Similar to VSG, licoricidin treatment had no significant effect on the thermogenic genes and protein levels in BAT (Figure S4A-B) and eWAT (Figure S4C-D). Collectively, these results demonstrate that a promoting role of licoricidin is beige fat thermogenesis in combating obesity in mice.

### Licoricidin promotes thermogenesis via the Adrb3-cAMP-PKA signaling pathway in adipocytes

**Figure 5:**
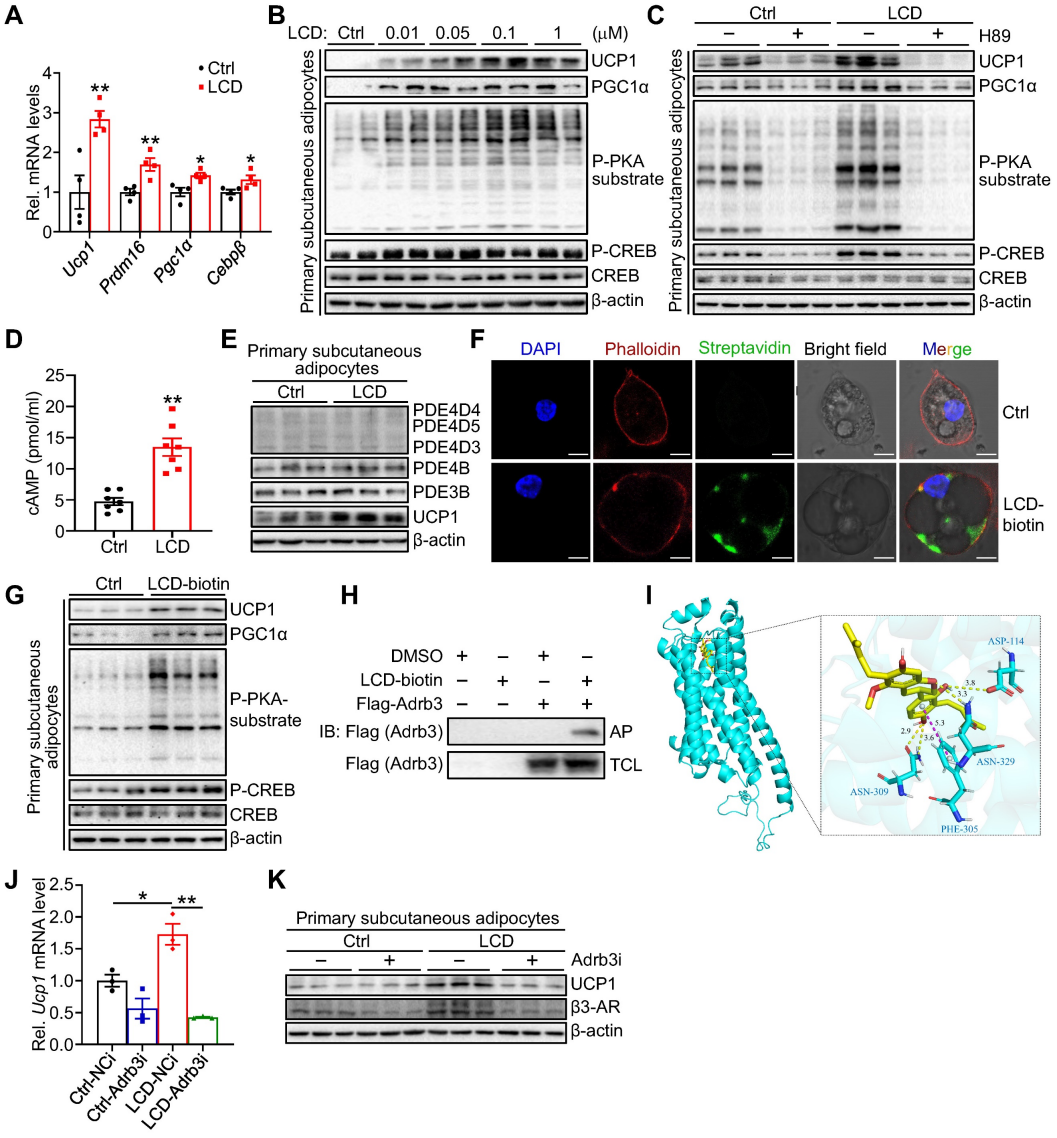
** Licoricidin promotes thermogenesis via the Adrb3-cAMP-PKA signaling pathway in adipocytes.** (A) The expression of thermogenic genes was measured by qPCR (*n* = 4 per group). Ctrl, control. (B) Differentiated primary subcutaneous adipocytes were treated with LCD for 24 h. The phosphorylation and protein level of CREB, phosphorylation of PKA substrate, and protein levels of UCP1, and PGC1α were analyzed by western blot. cAMP, cyclic adenosine monophosphate. CREB, cAMP response element-binding protein. P-CREB, the phosphorylation of CREB. PKA, protein kinase-A. P-PKA substrate, phosphorylation of PKA substrate. (C) Immunoblots (proteins indicated) were assessed in primary subcutaneous adipocytes incubated with or without PKA inhibitors H89 (10 µM) for 2 h and followed by LCD treatment for 24 h. (D) cAMP levels accumulated in primary subcutaneous adipocytes that were treated or untreated with LCD (0.1 µM) for 24 h (*n* = 7 per group). (E) Protein levels of PDE4D5, PDE4D4, PDE4D3, PDE4B, PDE3B were assessed by western blot following LCD (0.1 µM) treatment (*n* = 3 per group). PDE, phosphodiesterase. (F) Immunofluorescence images of primary adipocytes showed the position of LCD on the plasma membrane. Scale bar, 7 µM. (G) Immunoblots of indicated proteins in primary subcutaneous adipocytes treated or untreated with biotin-labelled LCD (0.1 µM) for 24 h (*n* = 3 per group). (H) Interaction between LCD and β3-AR was identified by immunoblot in 293T cells (*n* = 3 per group). IB, immunoblot. AP, affinity-pull down. TCL, total cell lysate. DMSO, dimethyl sulfoxide. (I) Representative images of autodocking for the transmembrane domain (TM) TM3, TM6, and TM7 of β3-AR and LCD. β3-AR, β3-adrenergic receptor. (J) Differentiated primary subcutaneous adipocytes were treated or untreated Adrb3 siRNA for 8h, followed by stimulated with LCD (0.1 µM) for 24 h. Ucp1 mRNA level was assayed by qPCR analysis (*n* = 3 per group). NCi, negative control-siRNA. Adrb3i, Adrb3-siRNA. (K) Immunoblot of UCP1 in differentiated primary subcutaneous adipocytes transfected (or not, -) with siRNA against Adrb3 (Adrb3i) (*n* = 3 per group). All data are representative of three independent experiments, each with a similar result. All data are means ± SEM. Statistical values *p < 0.05, **p < 0.01 are analyzed by Student's *t*-test.

To dissect the cellular mechanism by which licoricidin stimulates thermogenic gene expression, we treated primary subcutaneous adipocytes with licoricidin. Licoricidin treatment greatly increased the mRNA (Figure 5A) and protein (Figure 5B) expression of UCP1 and PGC1α as well as the phosphorylation of protein kinase A (PKA) substrates and cAMP-response element-binding protein (CREB) (Figure 5B). To determine whether licoricidin promotes thermogenic gene expression via the PKA signaling pathway, we treated primary subcutaneous adipocytes with licoricidin in the presence or absence of the PKA inhibitor H89. As we expected, H89 treatment greatly blocked licoricidin-induced phosphorylation of PKA substrates and CREB as well as the expression of UCP1 (Figure 5C). Licoricidin treatment greatly increased the cellular levels of cAMP in primary subcutaneous adipocytes (Figure 5D), suggesting that licoricidin may promote beige fat thermogenesis by acting at a site upstream of cAMP in the PKA signaling pathway. To elucidate the mechanism by which licoricidin activates PKA signaling, we first examined the potential effect of licoricidin on several cAMP-specific phosphodiesterases (PDEs), which have been shown to modulate cAMP levels in adipocytes 38,39. Relative to controls, however, licoricidin treatment had no effect on the expression levels of PDEs (Figure 5E). To determine how licoricidin could activate PKA signaling, we examined the cellular localization of biotin-labeled licoricidin by confocal microscopic experiments. We found that the biotin-labeled licoricidin, which could be detected by Alexa Fluor 488 streptavidin, is mainly localized at the plasma membrane of primary adipocytes (Figure 5F). Consistently, biotin-labeled licoricidin treatment could also greatly enhance the expression levels of several thermogenic proteins and activate PKA signaling pathway in primary subcutaneous adipocytes (Figure 5G). β-adrenergic receptors (β-ARs) located at the plasma membrane are key hubs in activation of PKA signaling pathway and the β3-AR is by far the predominant fashion in mice 40. To determine if licoricidin interacts directly with the β3-AR, we incubated lysates from the β3-AR-overexpressing cells with the biotin-labeled licoricidin or its control DMSO, followed by NeutrAvidin-agarose-bead pulldown. Licoricidin can interact with β3-AR, suggesting that β3-AR is a molecular target of licoricidin (Figure 5H). By bioinformatic analysis, we assessed the possible licoricidin binding sites on β3-AR. The predicted binding mode of licoricidin to the transmembrane domain (TM) revealed a good shape match between licoricidin and TM3, TM6 as well as TM7 (Figure 5I and Figure S5A). Moreover, the dissociation constant (Kd) and half-maximal effective concentrations (EC50) were measured as 9.446 μM and 8.471 μΜ in MST analysis, respectively, indicative of a good interaction between licoricidin and β3-AR (Figure S5B). To confirm an involvement of β3-AR in licoricidin action, we suppressed β3-AR expression in primary adipocytes by siRNA (Figure S5C). Suppressing β3-AR expression markedly inhibited licoricidin-induced UCP1 mRNA (Figure 5J) and protein (Figure 5K) expression. Taken together, these findings demonstrate that licoricidin promotes thermogenic genes expression in adipocytes by activating the Adrb3-cAMP-PKA signaling pathway.

### Licoricidin restrains the development of obesity and its metabolic consequences

**Figure 6:**
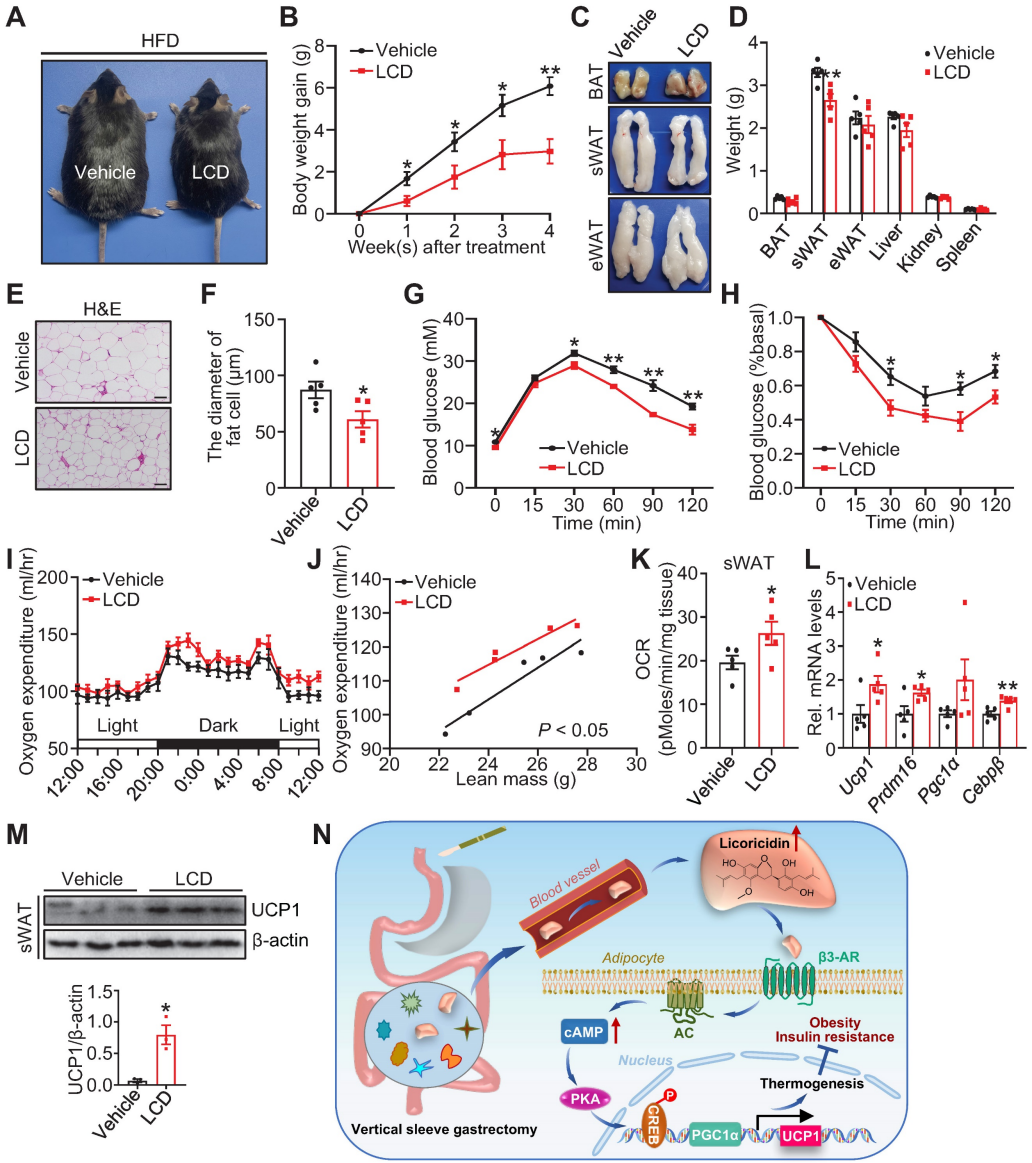
** Licoricidin restrains the development of obesity and its metabolic consequences.** (A) A representative image of HFD mice after LCD treatment. HFD mice were treated daily with LCD (10 µg/kg) by fad pad injection for 4 weeks (*n* = 5 mice per group). (B) Body weight gain of HFD mice during LCD treatment. (C) Representative photos of BAT, sWAT, and eWAT derived from vehicle and LCD mice. (D) Weights of sWAT, eWAT, BAT, and other tissues of vehicle and LCD mice (*n* = 5 mice per group). (E) H&E of sWAT-derived from vehicle and LCD mice. Scale bar, 100 µm. (F) sWAT adipocytes diameters in vehicle and LCD mice were quantified (*n* = 5 mice per group). (G) Glucose tolerance test was executed on vehicle and LCD mice (*n* = 5 mice per group). (H) Insulin tolerance test was executed on vehicle and LCD mice (*n* = 5 mice per group). (I) The oxygen consumption of vehicle and LCD mice (*n* = 5 mice per group) was examined. (J) The regression of oxygen consumption with lean mass in vehicle and LCD mice. (K) Basal OCR of isolated sWAT of vehicle and LCD mice was assayed by Seahorse (*n* = 5 mice per group). (L) The expression of thermogenic genes was measured by qPCR (*n* = 5 mice per group). (M) The protein level of UCP1 was detected by western blot. (N) A proposed model of the mechanism by which VSG combats obesity and insulin resistance. All data are means ± SEM. Statistical values *p < 0.05, **p < 0.01 are analyzed by Student's *t*-test or two-way ANOVA, or ANCOVA (J) between Vehicle and LCD groups.

To determine whether licoricidin has a direct effect on sWAT thermogenesis, we injected licoricidin (10 μg/kg) or vehicle into the subcutaneous fat pad of HFD-fed obese mice. Licoricidin injection significantly reduced mouse body size (Figure 6A) and weight (Figure 6B). Consistently, licoricidin treatment reduced the weight of sWAT, but not of eWAT, BAT, and other tissues (Figure 6C-D), concurrently with reduced adipocyte cell size in sWAT (Figure 6E-F). Licoricidin injection dramatically improved glucose tolerance (Figure 6G) and insulin resistance (Figure 6H) in mice. Licoricidin-treated mice had higher oxygen consumption and OCR than that of vehicle-treated mice (Figure 6I-K). Consistent with this result, the mRNA (Figure 6L) and protein (Figure 6M) levels of several thermogenic genes were greatly induced in sWAT of the licoricidin-treated mice compared to vehicle-treated mice. Licoricidin injection had no effect on the thermogenic gene or protein expression in BAT (Figure S6A-B) and eWAT (Figure S6C-D). Taken together, these results reveal that licoricidin has a direct and beige fat-specific effect on thermogenesis.

## Discussion

Bariatric surgery such as VSG has been recognized as the most effective intervention to counter obesity 8,41. While altered gut microenvironment including metabolites is implicated in mediating the beneficial effects of VSG 17,42. the mechanisms underlying the anti-obesity effects of VSG remain largely unclear.

In this study, we found that VSG in mice greatly increased the abundance of phylum *Firmicutes* and decreased the abundance of the detrimental bacteria such as *Dorea, Fusicatenibacter, Subdoligrenulum,* and* Bilophila* 37. In addition, we identified the gut metabolite licoricidin as a key molecule mediating the anti-obesity effect of VSG. VSG greatly increased serum licoricidin levels in mice. Intriguingly, licoricidin treatment by either oral gavage or fat pad injection alleviated obesity in mice, suggesting a therapeutic potential to combat obesity. Mechanistically, licoricidin stimulated thermogenic gene expression in adipocytes, which was mediated via binding to β3-AR and subsequent activation of the cAMP-PKA signaling pathway, a well-established signaling pathway to promote thermogenic gene expression. Collectively, these results uncover a previously undescribed signaling mechanism underlying the anti-obesity effect of VSG.

We found that licoricidin administration to mice by both oral gavage and fat pad injection effectively stimulated beige fat thermogenesis but had little effect on BAT thermogenesis. A possible explanation for this result is that adipose tissue is heterogeneous, and its function is dynamically orchestrated by the microenvironment within the tissue such as T cells and macrophages 43,44. Alternatively, given that both β3-AR and UCP1 are highly enriched in BAT 45,46, low doses of licoricidin may be insufficient to further increase BAT thermogenesis, demonstrating a beige fat-specific function of licoricidin in thermogenesis.

Licoricidin is an active compound found in licorice, one of the most commonly used herbal drugs in traditional Chinese medicine for the treatment of many diseases such as obesity, liver diseases, and tumors 47,48. The anti-obesity effects of licorice have been attributed mainly to glycyrrhizin, licochalcone A, and related derivatives 48-50. To the best of our knowledge, no information is currently available on whether licoricidin promotes thermogenesis and energy expenditure. In this study, we show that licoricidin alleviates obesity and insulin resistance in mice by promoting beige fat thermogenesis via activating the cAMP-PKA pathway in adipocytes, uncovering a signaling mechanism linking gut metabolite and adipose tissue by which VSG improves obesity and metabolism. While we found that VSG greatly increased licoricidin levels in the gut, it remains to be determined which gut microbiota plays an important role in licoricidin production. Nevertheless, the identification of licoricidin as an effective anti-obesity molecule offers a new opportunity for developing effective anti-obesity treatment.

In summary, we uncover a previously unidentified signaling mechanism by which VSG suppresses obesity in mice. In addition, we identify licoricidin as a critical gut metabolite that mediates the anti-obesity effects of VSG. Our findings point to the possibility of developing licoricidin-based therapeutic treatment for obesity and its associated metabolic diseases.

## Supplementary Material

Supplementary figures and table.

## Abbreviations

Adrbs: beta-adrenergic receptors
AP: affinity-pull down
BAT: brown adipose tissue
cAMP: cyclic adenosine monophosphate
CREB: cAMP response element-binding protein
Cebpβ: CCAAT enhancer binding protein β
CLAMS: comprehensive lab animal monitoring system
Ctrl: control
C-9-PA: Cis-9-palmitoleic acid
DMSO: methyl sulfoxide
DIO: diet-induced obese
Down: down-regulate
eWAT: epididymal white adipose tissue
FC: fold change
GTT: glucose tolerance test
HFD: high-fat diet
HPLC: high-performance liquid chromatography
1-O-2-OHSG-3-P: 1-o-hexadecyl-2-o-(5z,8z,11z,14z,17z-eicosapentaenoyl)-sn-glyceryl-3-phosphorylcholine
3-HC: 3-hydroxybutyryl carnitine
IB: immunoblot
ITT: insulin tolerance test
LCD: licoricidin
KEGG: Kyoto encyclopedia of genes and genomes
1-MSG-3-P: 1-myristoyl-sn-glycero-3-phosphocholine
5-5-M-1-PNOB: 5-(diethoxyphosphoryl)-5-methyl-1-pyrroline-n-oxide-biotin
NCD: normal chow diet
n.s.: not statistically significant
OCR: oxygen consumption rate
OPLS-DA: orthogonal projection to latent structure discriminant analysis
PDE: phosphodiesterase
PKA: protein kinase-A
PGC1α: peroxisome proliferator-activated gamma coactivator 1 alpha
Prdm16: PR domain-containing 16
qPCR: quantitative real-time PCR
sWAT: subcutaneous white adipose tissue
SM-5-T: S-methyl-5-thioadenosine
TM: transmembrane domain
TBG: Thymol-beta-d-glucoside
UCP1: uncoupling protein 1
Up: up-regulate
VSG: vertical sleeve gastrectomy
VIP: variable important for the projection
