# Supplementary material for: Vertical sleeve gastrectomy-derived gut metabolite licoricidin activates beige fat thermogenesis to combat obesity
Source: Theranostics. 2023 May 21;13(9):3103–16. doi: 10.7150/thno.81893 (PMC10240825; doi:10.7150/thno.81893)
Supplement: Supplementary file 1 — Supplementary figures and table. [file thnov13p3103s1.pdf]

Supplemental files

Vertical sleeve gastrectomy-derived gut metabolite licoricidin activates beige fat thermogenesis to combat obesity

Zhangliu Jin [1,*], Wen Meng [2,*], Ting Xiao [2,3], Jiangming Deng [2], Jing Wang [2], Jie Wen [2], Kai Chen [1], Liwen Wang [2], Juanhong Liu [2], Qingxin Li [2], Jieyu He [2], Zheng Wang [4], Wei Liu [1,✉], Feng Liu [2,✉]

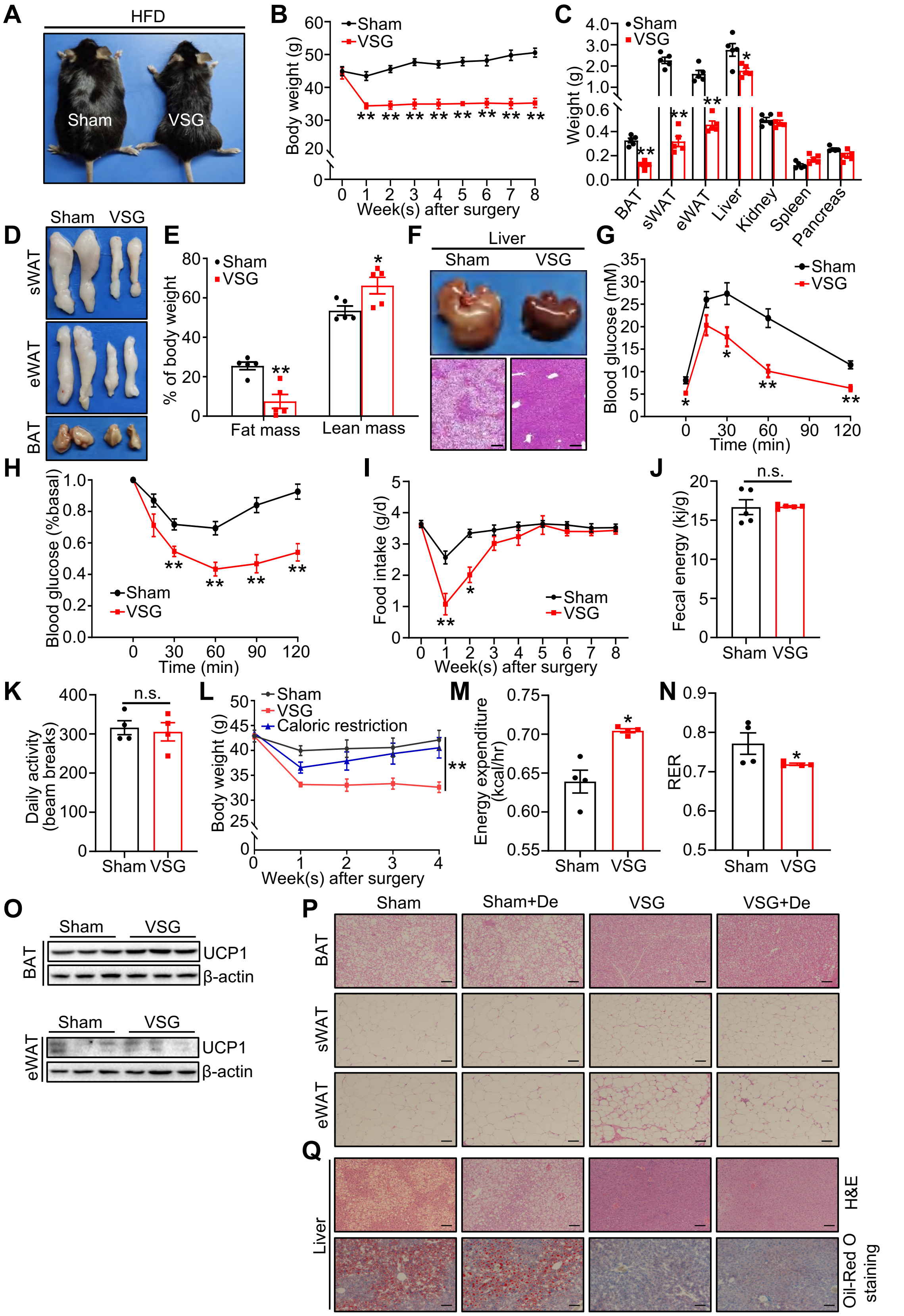

Figure S1. VSG resists HFD-induced obesity and insulin resistance (Related to Figure 1). (A) A representative image of VSG and Sham mice 8 weeks post-surgery. (B) Body weight of HFD mice during VSG treatment (n = 5 mice per group). (C) Weights of sWAT, eWAT, BAT, and other tissues of VSG and Sham mice (n = 5 mice per group) 8 weeks following surgery. (D) Representative photos of sWAT, eWAT, and BAT derived from VSG and Sham mice 8 weeks after surgery. (E) Body component of VSG and Sham mice 8 weeks post-surgery (n = 5 mice per group). (F) Representative images of H&E of liver-derived from VSG and Sham mice 8 weeks post-surgery. (G and H) Glucose tolerance test (G) and insulin tolerance test (H) was executed on VSG and Sham mice 4 weeks after surgery (n = 5 mice per group). (I) Daily food intake of mice from week 1 to week 8 following surgery (n = 5 mice per group). (J) Fecal energy of mice week 8 following surgery (n = 5 mice per group). ns, not significant. (K) Daily spontaneous physical activity of Sham and VSG mice week 8 post-surgery (n = 4 mice per group). (L) Body weight of Sham, VSG, and caloric restriction mice.* Sham vs. VSG. (M) The average of energy expenditure between Sham and VSG mice. (N) Respiratory exchange ratio (RER) between Sham and VSG mice. (O) UCP1 protein expression in BAT and eWAT of VSG and Sham mice 8 weeks post-surgery. (P) H&E of sWAT, eWAT, and BAT derived from Sham, Sham + De, VSG, and VSG+De mice. Scale bar, 100 µm. (Q) H&E and Oil-Red O staining of liver derived from Sham, Sham + De, VSG, and VSG + De mice. Scale bar, 100 µm. All data are means ± SEM. Statistical values *$p < 0.05$, **$p < 0.01$ are analyzed by two-way ANOVA, ANCOVA (M and N), or two-tailed unpaired Student's t-test between Sham and VSG groups.

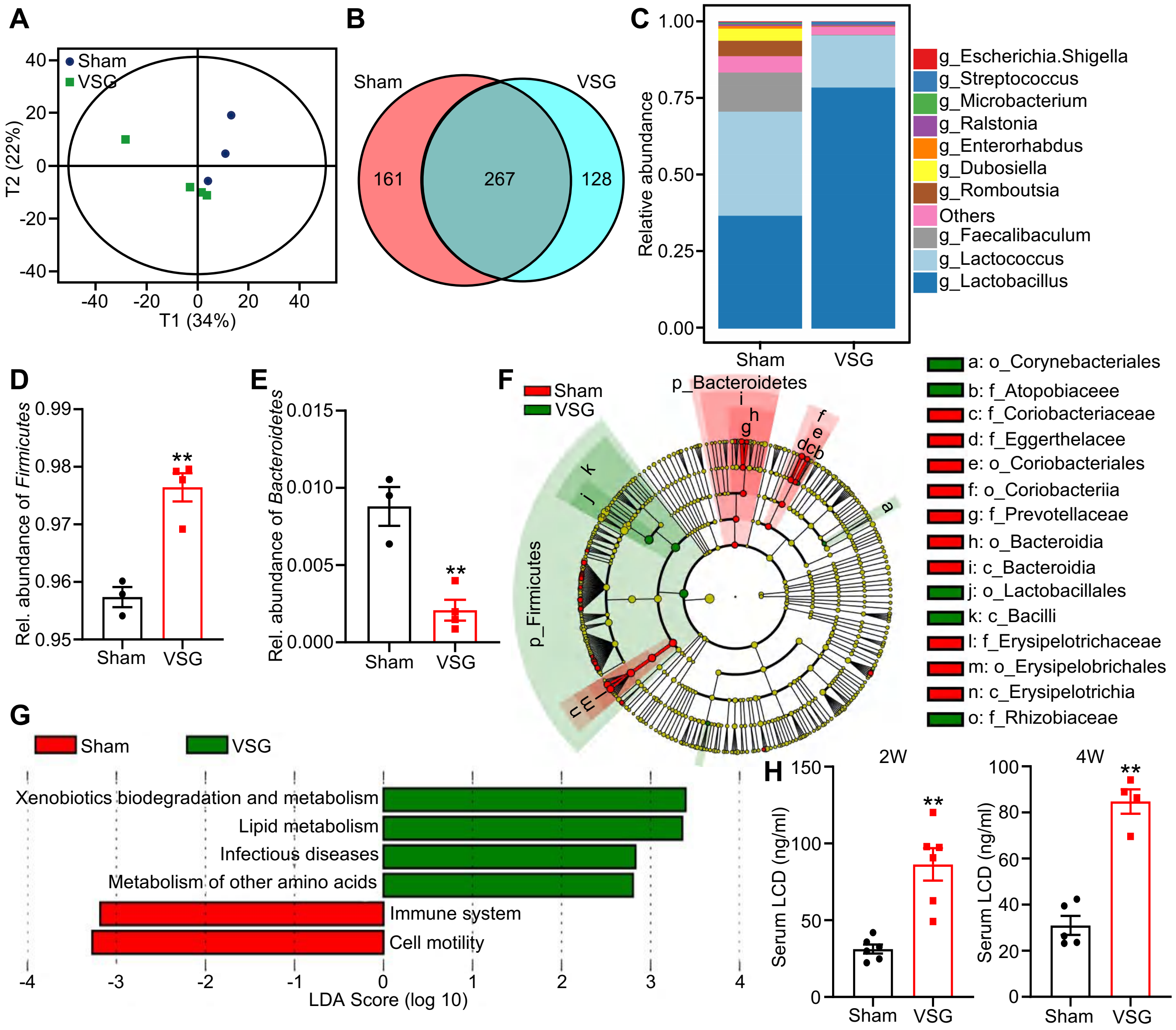

Figure S2. VSG alters intestinal microflora ingredients and metabolites (Related to Figure 2). (A) Principal component analysis between Sham (n = 3) and VSG (n = 4) groups using the Bray-Curtis distance. (B) Venn graph of operational taxonomic units (OTUs) from Sham (n = 3) and VSG (n = 4) groups. (C) Hierarchical clustering graph comparing feces of Sham (n = 3) and VSG (n = 4) mice. (D and E) The relative abundances of *Firmicutes* (D) and *Bacteroidetes* (E) were measured. (F) Taxonomic cladogram generated from LEfSe of 16S rRNA sequencing data. Red shows enriched taxa in the Sham group. Green shows enriched taxa in the VSG group. Each circle's size is proportional to the taxon's abundance. (G) Kyoto encyclopedia of genes and genomes (KEGG) function prediction displays the dominant functions from altered gut microbiota. (H) The LCD concentration in mouse serum was assessed at 2 and 4 weeks following surgery (n = 4-6 mice per group). All data are means ± SEM. Statistical values *p < 0.05, **p < 0.01 are analyzed by two-tailed unpaired Student's t-test or ANOVA between Sham and VSG groups.

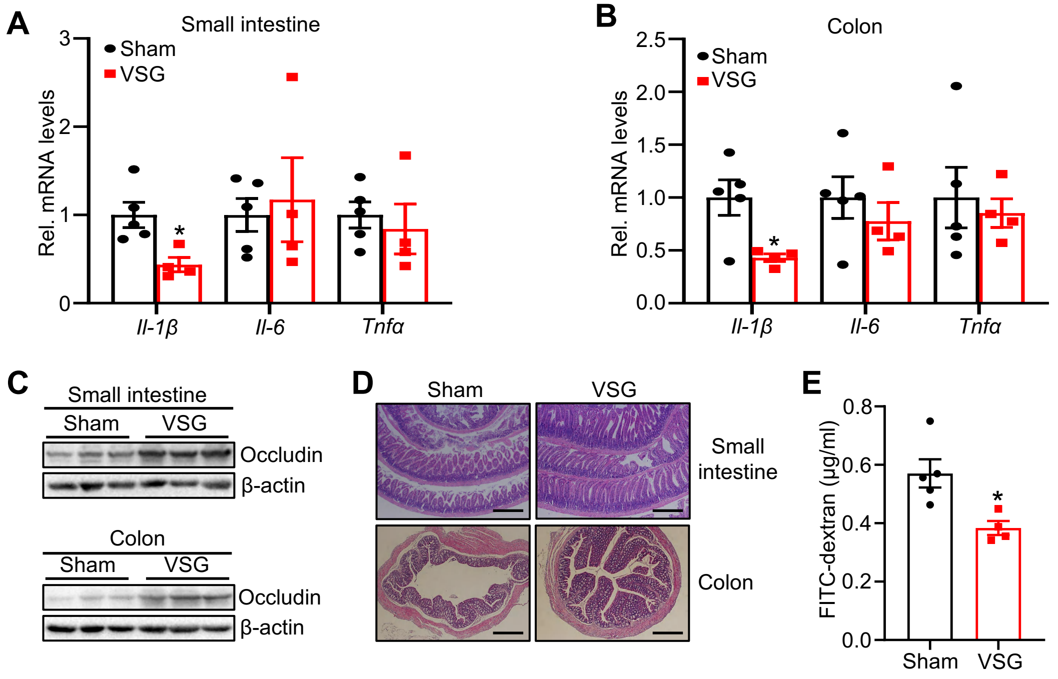

Figure S3. **VSG restores HFD-induced gut inflammation and barrier integrity (Related to Figure 2).** (A and B) mRNA levels of inflammatory cytokines in small intestine (A) and colon (B) of Sham (n = 5) and VSG (n = 4) mice 8 weeks after surgery were detected by quantitative real-time PCR (qPCR). (C) The levels of tight junction protein in small intestine and colon of VSG and Sham mice 8 weeks after surgery were measured by western blot. (D) Representative photos of H&E of small intestine and colon in VSG and Sham mice. Scale bar, 100 μm. (E) The intestinal permeability of the Sham (n = 5) and VSG (n = 4) group was measured via FITC-dextran oral gavage. All data are means ± SEM. Statistical values *p < 0.05, **p < 0.01 are analyzed by two-tailed unpaired Student's t-test between Sham and VSG groups.

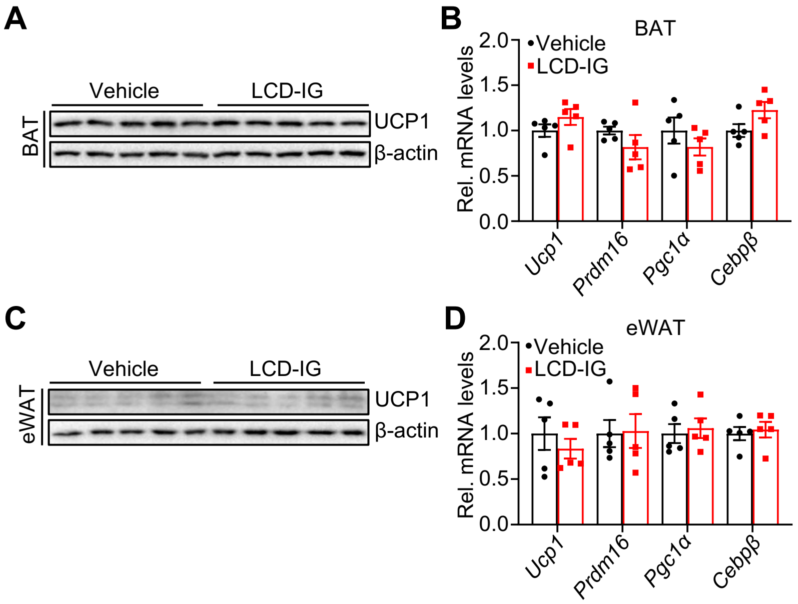

Figure S4. LCD treatment has no effect on promoting thermogenesis of BAT and eWAT (Related to Figure 4). (A-D) The protein levels of UCP1 and mRNA expression of thermogenic genes in BAT (A and B) and eWAT (C and D) of LCD-IG and vehicle mice after LCD intragastric administration (LCD-IG) were measured by western blot and qPCR (n = 5 mice per group). All data are means ± SEM. Statistical values $*p < 0.05$ are analyzed by two-tailed unpaired Student's t-test.

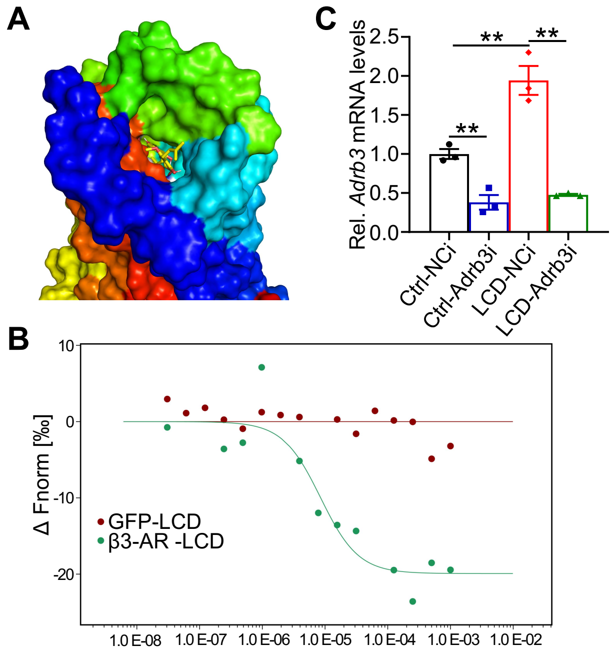

Figure S5. Licoricidin promotes thermogenesis via the Adrb3-cAMP-PKA signaling pathway in adipocytes (Related to Figure 5). (A) A representative image of autodocking for the transmembrane domain of β3-AR and LCD. β3-AR, β3-adrenergic receptor. (B) The affinity and half-maximal effective concentrations (EC50) between licoricidin and β3-AR were analyzed by the MST assay. (C) The Adrb3 mRNA levels after Adrb3-siRNA treatment (*n* = 3 per group). Data are representative of three independent experiments, each with a similar result. All data are means ± SEM. Statistical values $*p < 0.05$, $**p < 0.01$ are analyzed by two-tailed unpaired Student's *t*-test.

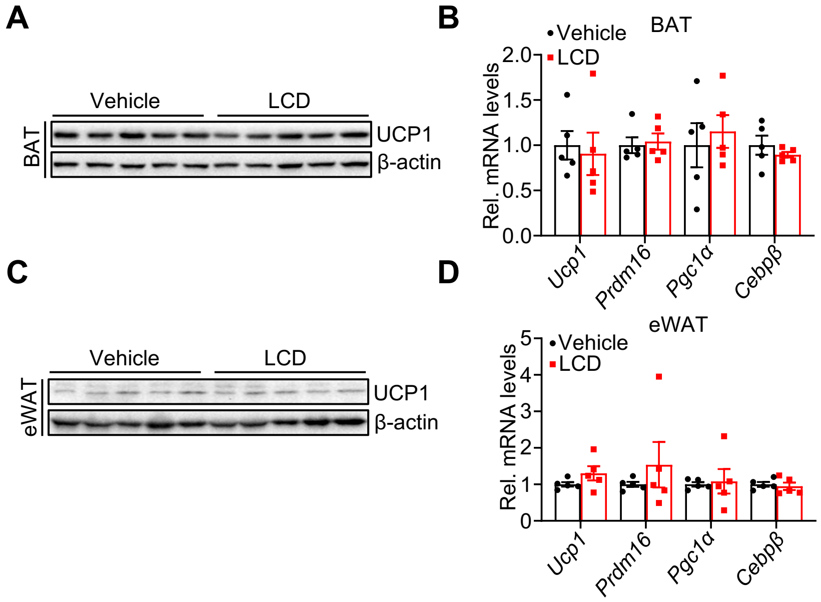

Figure S6. LCD treatment has no effect on promoting thermogenesis of BAT and eWAT (Related to Figure 6). (A-D) The protein levels of UCP1 and mRNA expression of thermogenic genes in BAT (A and B) and eWAT (C and D) of LCD and vehicle mice after LCD injected into sWAT were checked by western blot and qPCR (n = 5 mice per group). All data are means ± SEM. Statistical values *p < 0.05, **p < 0.01 are analyzed by two-tailed unpaired Student's t-test.

Table S1. Primer sequences for qPCR.

Gene	Forward primer (5'-3')	Reverse primer (5'-3')
Ucp1	GCTTTGCCTCACTCAGGATTGG	CCAATGAACACTGCCACACCTC
Prdm16	ATCCACAGCACGGTGAAGCCAT	ACATCTGCCCACAGTCCTTGCA
Pgc1α	GAATCAAGCCACTACAGACACCG	CATCCCTCTTGAGCCTTTCGTG
Cebpβ	CAACCTGGAGACGCAGCACAAG	GCTTGAACAAGTTCCGCAGGGT
Adrb3	AGGCACAGGAATGCCACTCCAA	GCTTAGCCACAACGAACACTCG
Il-1β	CCACAGACCTTCCAGGAGAATG	GTGCAGTTCAGTGATCGTACAGG
Il-6	AGACAGCCACTCACCTCTTCAG	TTCTGCCAGTGCCTCTTTGCTG
Tnfα	CTCTTCTGCCTGCTGCACTTTG	ATGGGCTACAGGCTTGTCACTC
β-actin	CATTGCTGACAGGATGCAGAAGG	TGCTGGAAGGTGGACAGTGAGG
